# Magnitude of Antenatal Depression and Associated Factors among Pregnant Women in West Badewacho Woreda, Hadiyya Zone, South Ethiopia: Community Based Cross Sectional Study

**DOI:** 10.1155/2020/2950536

**Published:** 2020-01-28

**Authors:** Mengistu Lodebo, Dagmawit Birhanu, Samuel Abdu, Tadele Yohannes

**Affiliations:** ^1^Department of Midwifery, Hossana College of Health Sciences, Hossana, Ethiopia; ^2^School of Nursing and Midwifery, Faculty of Health Sciences, Institute of Health, Jimma University, Jimma, Ethiopia; ^3^School of Public Health, College of Medicine and Health Sciences, Hawassa University, Hawassa, Ethiopia

## Abstract

**Background:**

Antenatal depression is prevalent and serious problems that is associated with psychosocial factors, obstetric history, and history of psychiatric illness. Evidence on prevalence and factors associated with antenatal depression at community level is limited in Ethiopia. The aim of this study was assessing the prevalence of antenatal depression and associated factors among pregnant women in West Badewacho Woreda, Hadiyya Zone, South Ethiopia, 2018.

**Methods:**

A community based cross sectional study was conducted from March 15 to April 12, 2018. To draw a total sample size of 541 pregnant women, multistage sampling technique was used. Pretested semi-structured questionnaire and standardized scale was used to collect data from each study subject. Data were entered and cleaned using Epi-Data version 3.1 and exported to SPSS version 23 for analysis. Bivariate analysis was carried out to see crude association between each independent variable and outcome variable. Odds ratios at 95%CI were computed to measure the strength of the association between the outcome and the independent variables. *P*-value < 0.05 was considered as statistically significant in multivariate analysis.

**Results:**

The prevalence of antenatal depression in the study population was 23.3% (CI: 19.8–26.8). Factors significantly associated with antenatal depression were marital status other than married (single, widowed, divorced) [AOR: (2.807; 95%CI: (1.268, 6.227); *p*-value = 0.042], history of previous depression [AOR: 3.414; 95%CI: (1.154, 12.999); *p*-value = 0.001] family history of mental illness [AOR: 3.874; 95%CI: (1.653, 7.052); *p*-value = 0.028], recent violence from intimate partner [AOR: 3.223; 95%CI: (1.359, 7.643); *p*-value = 0.008], unsatisfactory marital relation [AOR: 7.568; 95%CI: (3.943, 14.523); *p*-value < 0.001], lack of adequate social support [AOR: 5.491; 95%CI: (2.086, 14.451); *p*-value < 0.001] and unplanned current pregnancy [AOR: 2.013; 95%CI: (1.025, 3.953); *p*-value = 0.042].

**Conclusion:**

The prevalence of antenatal depression in west Badewacho woreda was high and it is associated with marital status, unplanned current pregnancy, history of previous depression, family history of mental illness, recent violence from intimate partner, poor marital satisfaction level, and poor social support. Improving maternal and child health services and introducing screening for depression as part of routine antenatal assessment to curb antenatal depression should get due attention.

## 1. Introduction

Pregnancy and the transition to motherhood comprise major physical and psychological changes in the expecting mothers. These changes can become associated to the development of depression during the pregnancy [1]. Depression is a common mental disorder that presents with depressed mood, loss of interest or pleasure, decreased energy, feelings of guilt or low self-worth, disturbed sleep or appetite, and poor concentration. It is a significant contributor to the global burden of disease and affects people in all communities across the world. The burden of depression is 50% higher for females than males. In fact, depression is the leading cause of disease burden for women in both high-income and low- and middle-income countries [2].

Antenatal depression is one of maternal depression that starts during pregnancy. Depression during pregnancy can be very distressing and hard to predict. Between 10 and 15 percent of pregnant mothers experience mood swings during pregnancy that last more than two weeks at a time and hinder with normal day-to-day functioning. The signs and symptoms of depression during pregnancy are similar to those of depression in any other time [3, 4].

Globally, 322 million people were living with depression in 2015; this represents over 4% of the global population. In the World Health Organization African Region 53 million people suffer from depression. Depression is more common among females (5.1%) than males (3.6%). There are effective treatments for depression but nearly 50% of people with depression do not get treatment. Low levels of recognition and access to care for depression lead to an estimated global economic loss of more than a trillion US dollars every year [5, 6].

Globally, pregnant women experiences about 10% of mental disorders, primarily depression. In developed countries 10–15% of women, and developing countries between 20 and 40% of women experience depression during pregnancy or after childbirth. Mental health issues are more common than other medical and obstetrical problems that women are routinely screened and treated for in pregnancy. Antenatal depression has been identified as a serious health problem, but is a neglected component of care for women during pregnancy [5, 7, 8].

Women living in developing countries are more exposed to risk factors, which increase their susceptibility to develop mental health problems [7]. Like poor socioeconomic status, less valued social roles, and status, unintended pregnancy, intimate partner violence, rape, Pre-existing psychological disturbances often surface as depression, substance abuse, or attempts at suicide. Presence of chronic medical conditions prior to pregnancy, preeclampsia, hypertension, and gestational diabetes related to antenatal depression [9, 10].

There are many opportunities for pregnant women to be identified and treated because they are in frequent contact with universal services (maternity, health visiting, and primary care) for self and their baby's care. Despite the fact that the pregnant women and new mothers experiencing depression often do not get the treatment they need due to fear of discussing mental health concerns with their providers or a lack of education about depression [11, 12].

Generally, antenatal depression is a public health issue due to its adverse consequence on the overall health of the woman and its relation with low utilization of antenatal care services, complications during pregnancy, bad experience of childbirth, adverse pregnancy outcomes like still birth, low birth weight, preterm birth, and birth asphyxia), and infant mortality, and morbidity (i.e., poor infant growth and development) [13–15].

Though depression is a common medical disorder during pregnancy, it is persistently under diagnosed and under treated during antenatal care and is largely ignored especially in developing countries despite locally available and affordable interventions. A systematic review reported that 16% of pregnant women experience depression in low- and middle-income countries. High rates of mental health problems in pregnant women have been reported from many countries in Africa such as Ethiopia, Nigeria, Senegal, South Africa, Uganda, Zimbabwe, and many others [4, 16].

In Ethiopia more than one in ten pregnant women suffers from undetected depression. Around half of those affected by depression have thoughts of ending their life [17].

Different studies show that the prevalence of antenatal depression is increasing in globe. However, attention to antenatal depression is still low. This may be due to there being no evidence-based guidelines regarding recommended intervals (i.e., timing or frequency) for antenatal depression screening, and the optimal settings, tools, and targets. Research conducted in area of antenatal depression in different parts of the world focus on facility, including in Ethiopia. Determining the prevalence and associated factors with antenatal depression in community level is important to early identify and manage the problem. So that, the study finding is important to health professionals, who are working in an antenatal care room capable to identify pregnant mothers at risk of developing antenatal depression and counsel them. It is also helpful for health extension workers to recognize antenatal depression as a health problem, identify, and support the women who are at risk of depression and give special attention for them by educating the women and husband. The study finding will help health facilities to give attention for depression during pregnancy and diagnosis and manage early before complications arise.

Finding of this study is also important as input for policy makers and experts to develop policy and strategies to antenatal depression screening. Other researchers will be used as a milestone for further research in the study area.

Furthermore, as the investigators search, there were no previously published and conducted study in Hadiyya zone as a general as well as in west Badewacho woreda. So, the aim of this study was to assess prevalence and associated factors among pregnant women in West Badewacho Woreda, Hadiyya Zone.

## 2. Methods

### 2.1. Study Area

West Badewacho woreda is one of the 10 Woredas (districts) in Hadiyya Zone, Southern Nations, Nationalities and Peoples Region (SNNPR). It is located about 357 km south west of the Capital city of the country and 127 km from the regional Capital, Hawassa. The average elevation is 1756–2000 m above sea level. In the Woreda there are four health centers, and 22 health posts that provide basic health care. According to 2010 Ethiopian fiscal year district population estimation, total population of the woreda was 108,164 with total households of 22,074. There were 25,202 reproductive age groups, of which 3742 were pregnant women.

### 2.2. Study Design and Period

A community based cross sectional study was conducted from March to April, 2018.

### 2.3. Study Participants

**Figure 1:**
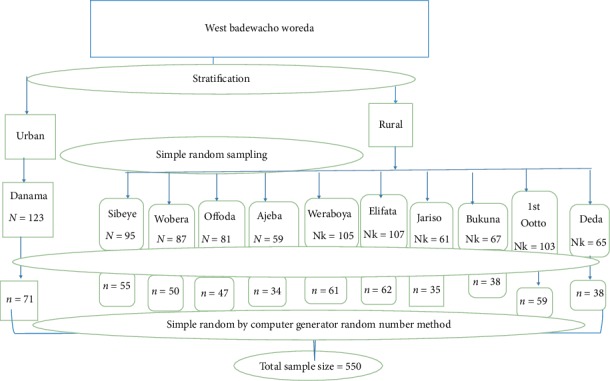
Schematic presentation of sampling procedure of randomly selected kebeles of West Badewacho Woreda, 2018.

The source population of the study was all pregnant women in west Badewacho woreda. The study population was all pregnant women in selected kebeles in west Badewacho woreda who fulfill the inclusion criteria Figure 1. All pregnant women registered by health extension workers were included in the study and Pregnant women who were critically ill, unable to speak and listen were excluded from the study.

### 2.4. Sample Size Determination and Sampling Technique

Sample size was determined using Epi info version 7 for single population proportion by considering Proportion of antenatal depression from community based study conducted in Debra Tabor Town which was 11.8% [18]. Confidence level of 95% and 0.04 margin of error were taken in the calculation. Considering 10% no response rate and design effect 2, the determined sample size was 550.

Multistage random sampling technique was employed to select the study subjects. First, stratification of woreda into urban and rural kebeles, then all the rural and urban Kebeles in the woreda was listed separately. Then, to make 50% representative, ten rural kebeles from the twenty rural kebeles to one urban kebeles from two urban kebeles were selected by simple random sampling technique using lottery method. Then proportional allocation of sample size was done to each randomly selected kebeles based on six-month performance of kebeles. Participants were identified by obtaining official lists of the pregnant women from health extension workers of the randomly selected kebeles, who routinely collect data on new pregnancies to make sampling frame. Pregnant women were selected using simple random sampling from the existing sampling frame of pregnant women using computer generating random number method. Data collectors went to the home of the participants by using name and “gotti” number with guidance of health extension workers.

### 2.5. Data Collection and Measurement

There were four parts of instruments/tools for this study:

#### 2.5.1. Part 1 Structures and Semi-Structured Questionnaire

Structured and semi structured questionnaires consist of 31 items: 8 items about socio demographic characteristics, 11 items includes obstetric factors, 4 items for psychiatric and chronic illness history factors, 4 items for psychosocial factors, and 4 items for substance use history factors.

#### 2.5.2. Part 2 Edinburgh Postnatal Depression Scale

To assess antenatal depression the Edinburgh Postnatal Depression Scale (EPDS) was used due to it omits the somatic symptoms that are often associated with depression and that may be confounded by the changes associated with pregnancy like changes in appetite or weight, increased nausea, and headaches. The EPDS is a 10 item questionnaire, scored from 0 up to 3 (higher score indicating more depressive symptoms), that has been validated for detecting depression in ante partum and postpartum samples in different parts of the world. The instrument was validated in public health centers in Addis Ababa for postpartum use and showed sensitivity of 84.6% and specificity of 77.0% [19]. Like other similar studies conducted abroad and in Ethiopia, cutoff point of EPDS was 13 to identify pregnant women with depressive symptom [13, 15]. The pretest finding of EPDS for Cronbach's alpha was 0.854 which is acceptable since a reliability coefficient of 0.70 or higher is considered “acceptable” in most behavioral science research.

#### 2.5.3. Part 3 Social Support Level

Social support level received by women was measured by the Oslo3-item Social Support Scale (OSS) [20]. Its pretest finding for Cronbach's alpha was 0.757 which is acceptable.

#### 2.5.4. Part 4 Marital Satisfaction Level

Quickly marital satisfaction was assessed by Kansas marital satisfaction scale which is 3 items, each of the items on a 7 point scale ranging from one (extremely dissatisfied) to 7 (extremely satisfied). The scale has 21 sum score with minimum of 3 scores [21]. Its pretest finding for Cronbach's alpha was 0.964 which is also acceptable.

### 2.6. Data Collection Method

Seven diploma nurses were recruited as data collector while three BSc nurses were recruited as supervisors. The principal investigator was followed overall data collection procedure.

Face to face interviewer administered method of data collection was employed using Hadiyisa language version of structured and semi structured questionnaire.

### 2.7. Quality Assurance

Two days training was provided to the data collectors and supervisors on the data collection tool, interview technique, eligible study subjects, sampling techniques, and consent by principal investigator, and psychiatric nurse. The questionnaire was first prepared in English, translated into Hadiyisa, and then it was re-translated back to English to check for its consistency. Before a week of actual data collection investigator, supervisors, and data collectors took a 5% (28 sample) pre-test of the Hadiyisa version of questionnaire in East Badewacho Woreda Buligita and Mazoria kebeles similar characteristics to the study population to ensure clarity of the questionnaire and then the necessary modifications and correction was made to standardize and ensure its validity.

Supervision was conducted on daily basis for completeness and consisted of the filled questionnaires. Then, necessary feedback was offered to data collectors the next morning before commenced data collection. In addition, the data were thoroughly cleaned and carefully entered in to computer for analysis.

### 2.8. Data Processing and Analysis

Data were coded and entered into Epi-data version 3.1 and finally exported to SPSS version 23.0 statistical software for analysis. Recoding and computing of variables were done as needed. Descriptive statistic such as frequency, percentage, mean, standard deviation, and range were used to summarize the data. Bivariate analysis was done to check crude association between the outcome and independent variables. All variables which had a significant association at *p*-value ≤ 0.25 in the bivariate analysis were taken to multivariate analysis to include all potential variables. Back ward likelihood ratio of logistic regression was performed to identify the factors associated with antenatal depression. The model goodness fit was checked by Hosmer–Lemeshow test and the *p*-value was found to be 0.508 (>0.05), which revealed as the model was good. Odds ratios (AOR) at 95%CI were computed to measure the strength of the association between the outcome and the explanatory variables. *P*-value < 0.05 was considered as statistically significant in multivariate analysis.

## 3. Results

### 3.1. Socio-Demographic Characteristics of Respondents

From the total of five hundred fifty women, 541 women completed the interview which gave response rate of 98%.

In this study, the age of participants ranged from 18 to 45 years. The mean age of respondents was 27.28 years (Standard Deviation, SD = 4.758). Two hundred thirty one (42.7%) of study participants were in the age range of 25–29 years. Four hundred seventy (86.9%) of participants were rural residents. Regarding occupational status, 321 (59.3%) were housewives. One hundred sixty eight (31.1%) of the women attended secondary education. Concerning religion, 403 (74.5%) respondents were protestant religion followers. Nearly two third, 341 (63%) of the respondents were Hadiyya ethnic group. Regarding marital status, 465 (86%) of pregnant women were married. Three hundred ninety five (73%) of study participants had an average family monthly income below 1000 Ethiopian birr (Table 1).

### 3.2. Obstetric Characteristic of the Respondents

From total study participants, more than half 309 (57.1%) of the respondents were in third trimester pregnancy followed by second trimester 214 (39.6%). Four hundred thirty four (80.2%) were multigravida. Two hundred ninety seven (68.4%) of the respondents were multipara and the rest were primipara 107 (19.8%). Forty four (10.1%) women reported that they had history of abortion. Thirty two (7.4%) respondents had history of stillbirth. Regarding previous mode of delivery, majority of women 384 (88.5%) delivered spontaneously.

Fifty nine (13.6%) women had history of one or more complication during last pregnancy, labor, and delivery. Of this, prolonged labor, 29 (5.4%), and hyperemesis gravidarum, 22 (4.1%). Regarding complication in current pregnancy, 43 (7.9%) of respondents experienced current pregnancy complications. Almost three fourth, 413 (76.3%) of the women planned their current pregnancy. Out of 434 parity, 44 (10.1%) had history of abortion, and 32 (7.4%) had history of still birth (Table 2).

#### 3.2.1. Psychiatric and Medical Illness Characteristic of the Respondents

According to this study finding, 106 (19.6%) of women reported that they experienced depression previously. Thirty (5.5%) of respondents had family history of mental illness. From women interviewed for taking any drugs currently for other medical illness, 15 (2.8%) of women responded that they were using drugs currently. Types of medical illnesses were hypertension, 2 (13%), Diabetic mellitus, 2 (13%), anemia, 5 (30%), and others like kidney failure, and asthma, 6 (56%).

#### 3.2.2. Psychosocial Characteristic of Respondents

Sixty two (11.5%) of the respondents had experienced recent violence from their intimate partner such as physical 20 (3.7%), psychological 20 (3.7%), Psychological and physical 14 (2.6%), and sexual 17 (3.1%). Nearly half, 263 (48.6%) of the pregnant women received strong social support, 168 (31.1%) received moderate social support, and 110 (20.3%) received poor social support. Regarding marital relation satisfaction 96 (20.6%) of the respondents were not satisfied in marital relation. Six (1.1%) of mother reported that they encountered family member death. Ten (1.8%) of the participants reported that there was current family member sickness.

### 3.3. History of Substance Use amongst Participants

In this study, both mother and husband were interviewed for substance use history in current pregnancy until data collection period. Twenty one (3.9%) of the mother had history of substance use in current pregnancy. Eighty one (17.4%) of husbands used substances such as alcohol 57 (10.5%), Khat 27 (5%), and cigarette 22 (4.1%).

#### 3.3.1. Prevalence of Antenatal Depression

**Figure 2:**
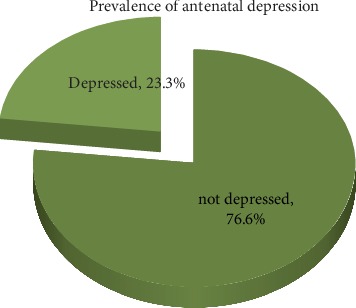
Prevalence of antenatal depression among pregnant women in West Badewacho Woreda, Hadiyya zone, South Ethiopia, 2018 (*n* = 541).

From a total of 541 study participants, 126 (23.3%) mothers were depressed (Figure 2). Sixty eight (12.6%) of participants were depressed at third trimester. 56 (10.4%) and 2 (0.4%) of women depressed at second and first trimester respectively. From total of 541 study participants, 12 (2.2%) women reported suicidal thoughts.

### 3.4. Interpretation Classification of Antenatal Depression

According to this study findings, 345 (63.8%) of mothers recognized as depression not likely, 46 (8.5%) of study participants had possible depression, 47 (8.7%) of respondents had fairly high possibility of depression, and 103 (19%) of study participants had probable depression.

#### 3.4.1. Factors Associated with Antenatal Depression

Selected variables that were significantly associated at the bivariate analysis were further examined in the multivariate logistic regression to see their relative effects on antenatal depression. Result of bivariate analysis showed that work status of the women, educational status of the women, marital status of the women, family monthly income, current pregnancy status, history of previous depression, family history of mental illness, recent violence from intimate partner, marital satisfaction level, social support level, substance use by mother, and substance use by husband as candidates for multivariate analysis (Table 3).

In multivariate logistic regression analysis marital status, current pregnancy status, history of previous depression, family history of mental illness, recent violence from intimate partner (husband), marital satisfaction level, and social support level were significantly associated with antenatal depression (Table 4).

The result showed that the likelihood of antenatal depression was about 3 times higher in marital status category of (single, widowed, divorced)) as compared to married women [AOR: 2.807; 95%CI: (1.268, 6.227)]; *p*-value = 0.042]. Those women who had not planned their current pregnancy were 2 times more likely to have antenatal depression as compared to those who had planned their current pregnancy [AOR: 2.013 (1.025, 3.953); *p*-value = 0.042]. The pregnant women who had history of previous depression were about 3 times more likely to experience antenatal depression when compared to their counter parts [AOR: 3.414; 95%CI: (1.154, 12.999); *p*-value = 0.001]. The odds of antenatal depression was about 4 times higher among pregnant women who had family history of mental illness as compared to women who reported no family history of mental illness [AOR: 3.874; 95%CI: (1.653, 7.052); *p*-value = 0.028]. Pregnant mothers who reported that there was recent violence from intimate partner (husband) had about 3 times higher odds to experience antenatal depression when compared with mothers who reported no recent violence from intimate partner [AOR: 3.223; 95%CI: (1.359, 7.643); *p*-value = 0.008].

The likelihood of experiencing antenatal depression among those who are not satisfied in their marital relation was nearly 8 times higher than among those satisfied in their marital relation [AOR: 7.568; 95%CI: (3.943, 14.523); *p*-value < 0.001]. Similarly, those who received poor social support were about 5 times and moderate social support were about 6 times more likely to experience antenatal depression as compared to women who received strong social support [AOR: 5.491; 95%CI: (2.086, 14.451); *p*-value = 0.001] and [AOR: 5.788; 95%CI: (2.361, 14.138); *p*-value < 0.001] respectively (Table 4).

## 4. Discussion

This study attempted to determine prevalence and factors associated with antenatal depression in West Badewacho Woreda, Hadiyya Zone.

According to this study, the prevalence of antenatal depression was 23.3% (95%CI: 19.8, 26.8). This study finding was higher when compared with study finding in Debra tabor town which reported that 11.8% of the study participants were depressed [18]. The discrepancy might be due to difference in sampling technique and setting. The Debra tabor study used cluster-sampling technique while current study used multistage sampling technique. Debra tabors study conducted on urban setting only while current study conducted on rural and urban. Also this study finding was higher when compared with study findings in Malaysia (13.8%) [22] and Ghana (9.9%) [23]. This discrepancy might be because of the difference in study design, study setting, sample size, inclusion criteria, social support practice, socio-demographic, and cultural characteristics.

The prevalence of current study is somewhat similar with findings of study conducted in Nigeria (24.5%) [24], Gondar university hospital (23%) [25], and Addis Ababa (24.94%) [26]. However, it is lower when compared with the study findings of Saud Arabia which reported 54.5% study participants who were depressed [27]. The variation might be due to difference in setting, sample size, and diverse socio economic position. Similarly, the study finding in Maichew Town, Ethiopia which showed 31.1% were depressed was higher compared with current study finding [28]. Sample size differences, setting, different screening tool, and sampling procedure might contribute to this variation. Other studies conducted in Pakistan (29.7%) [29], South Africa (38.5%) [30], and Northern Tanzania (33.8%) [13] also reported higher prevalence of antenatal depression when compared with the current study findings. This difference in the prevalence rates of antenatal depression reported by various studies might be attributed to social norms, cross cultural differences, socio economic differences, sample size, and research reporting systems.

The factors independently associated with antenatal depression were marital status, current pregnancy status, history of previous depression, family history of mental illness, recent violence from intimate partner (husband), marital satisfaction level, and social support level. Marital status category of (single, divorced, and widowed) were 2.8 times more likely to be depressed during antenatal period as married women. This is in agreement with findings of South Africa [30], two studies of Nigeria [24, 31], and Ethiopia [28]. This might be due to lack of support from families and male partners, having unwanted pregnancy, and poor financial capacity. In addition, in a traditional Ethiopia setting, any woman who becomes pregnant while not having husband is viewed as promiscuous and single parenting is socially unacceptable. The stigma associated with this may contribute to depressed moods.

Unplanned pregnancy was associated with antenatal depression. Those women who had not planned their current pregnancy were 2 times more likely to have antenatal depression as compared to those who had planned their current pregnancy. This is consistent with study findings in Malaysia [22], Brazil [32], and two cross sectional studies in Ethiopia which were done in Addis A baba [26] and Debra tabor town [18]. This study also found that history of previous depression was strongly associated with antenatal depression. The pregnant women who had history of previous depression were around 3 times more likely to experience antenatal depression as compared to their counter parts. This finding is in line with studies conducted in India [33], Brazil [34], South Africa [30, 35], and in Ethiopia [18, 26]. This might be due to those pregnant women were more biologically at risk to depression, and the hormonal changes of pregnancy increase vulnerability to depression, or their psycho-social context may make them vulnerable to recurrent depression.

The current study also identified that the odds of antenatal depression was about 4 times higher among pregnant women who had family history of mental illness as compared to women who reported no family history of mental illness. This is in line with studies conducted in Ethiopia on perinatal women [36, 37] but contradicting with study finding in Pakistan [38]. The variation might be due to the differences of methodology (inclusion criteria) and analysis method which is only square. This might not show direction, strength of association, sociocultural, and cut point of the tool for antenatal depression.

The findings of this study indicated that pregnant mothers who reported recent violence from intimate partner (husband) had 3 times higher odds to experience depression as compared to mothers who reported no recent violence from intimate partner. This might be due to violence being inherently humiliating, especially during the reproductive life when the escape routes are often reduced. This humiliation can effect in the onset of depression, considering the social theory of origin of this disorder proposed by Brown and Harris in 1978 which argues that depression is a consequence of the experience of humiliation and imprisonment by an individual [39]. Based on this, the findings of the present study might find reason in the assumption that the history of violence is a generator of sadness and distress for pregnant women, when they recall the humiliation suffered. This finding was supported by findings of rural Bangladesh [40] and Malawi [41].

Another strong predictor for antenatal depression was marital satisfaction level. The likelihood of experiencing antenatal depression among those who are not satisfied in their marital relation was nearly 8 times higher as compared to the satisfied one. The current study finding was congruent with findings from Greek [42], Northern Tanzania [13], and rural Bangladesh [40]. This might be due to that pregnant women may prefer to share pregnancy worries only with their partners rather than their social network. Social support level was significantly associated with experiencing of antenatal depression. In this study the mother who received poor and moderate social support were more likely to experience antenatal depression when compared with women who received strong social support. This study finding is congruent with the study findings of South Korea [43], Nigeria [31], Malawi [41], and Ethiopia [26]. Receiving social support from friends and relatives during stressful times is thought to be a protective factor against developing depression. In addition, those women who receive support during their pregnancy may be more empowered to deal with their pregnancy and their home responsibility.

### 4.1. Limitation of the Study

As being cross-sectional in the design, this study lacks to show temporal relationship between cause and effect. Moreover, the mothers may commit social desirability bias to avoid stigma that could follow stated mental health problem and recall bias for some of the assessed characteristics, like history of previous depression. In addition, this study failed to assess hormonal and genetic factors that may importantly determine antenatal depression.

## 5. Conclusion

The finding of this study revealed that more than one in five respondents was depressed during pregnancy. Marital status, unplanned current pregnancy, history of previous depression, family history mental illness, recent violence from intimate partner, poor marital satisfaction level and poor social support were factors independently associated with antenatal depression. Introducing screening for depression as part of routine antenatal assessment, strengthening existing early antenatal visit and intervention measures, linking maternal mental health care services during antenatal period, establishing social support network, and health education to prevent unplanned pregnancy should get due attention. Further longitudinal prospective studies are also needed to fully understand the nature of these factors in antenatal depression.

## Figures and Tables

**Table 1:** Socio-demographic characteristic of respondents in West Badewacho Woreda, Hadiyya zone, South Ethiopia, 2018.

Variables	Categories	Frequency	Percentage
Age	15–19	13	2.4
	20–24	142	26.2
	25–29	231	42.7
	30–34	110	20.3
	>35	45	8.3
	Total	541	100.0

Residence	Urban	71	13.1
	Rural	470	86.9
	Total	541	100.0

Work status	Farmer	10	1.8
	Government employee	55	10.2
	Merchant	84	15.5
	Daily laborer	47	8.7
	Housewife	321	59.3
	Others	24	4.4
	Total	541	100.0

Others: students, non-government employers			
Educational status	Cannot read and write	119	22.0
	Read and write	68	12.6
	Primary	131	24.2
	Secondary	168	31.1
	Higher or tertiary	55	10.2
	Total	541	100.0

Religion	Orthodox	43	7.9
	Catholic	71	13.1
	Muslim	22	4.1
	Protestant	403	74.5
	Others	2	.4
	Total	541	100.0

Others: only Jesus, Adventist			
Ethnicity	Hadiyya	341	63.0
	Kambata	105	19.4
	Wolayita	53	9.8
	Gurage	17	3.1
	Sidama	4	.7
	Silte	17	3.1
	Others	4	.7
	Total	541	100.0

Others: Tigre, Ahmara, Oromo			
Marital status	Married	465	86.0
	Single	14	2.6
	Divorced	27	5.0
	Widowed	35	6.5
	Total	541	100.0

Average family monthly income(Eth. Birr)	<1000	395	73.0
	1001–2000	56	10.4
	>2001	90	16.6
	Total	541	100.0

**Table 2:** Obstetric characteristic of the respondents in West Badewacho Woreda, Hadiyya zone, South Ethiopia, 2018.

Variables	Categories	Frequency	Percentage
Pregnancy by trimester (*n* = 541)	First trimester	18	3.3
	Second trimester	214	39.6
	Third trimester	309	57.1

Gravidity (*n* = 541)	Primigravida	107	19.8
	Multigravida	434	80.2

Parity (*n* = 434)	Primipara	137	31.6
	Multipara	297	68.4

Previous mode of delivery (*n* = 434)	Spontaneous vaginal	384	88.5
	Cesarean section	50	11.5

Types of complication during last pregnancy, labor, and delivery	Hypertensive disorder	7	1.3
	Antepartum bleeding	18	3.3
	Hyperemesis gravidarium	22	4.1
	Prolonged labor	29	5.4
	Obstructed labor	5	0.9
	Mal presentation	6	1.1
	Postpartum hemorrhage	8	1.5
	Newborn complication	18	3.3
	Others^∗^	8	1.5

Types of complication in current pregnancy	Preeclampsia	7	1.3
	Hyperemesis gravidarum	21	3.9
	Malaria infection	9	1.7
	Others^∗∗^	6	1.2

Others: ^∗^retained placenta, ^∗^premature rupture of membrane, ^∗^preterm labor, ^∗^gestational diabetic mellitus, ^∗^post term, ^∗∗^malpresentation, ^∗∗^eclampsia, ^∗∗^antepartum hemorrhage.

**Table 3:** Bivariate analysis of antenatal depression and associated factors among pregnant mothers in West Badewacho Woreda, Hadiyya zone, South Ethiopia, 2018.

Variables	Categories	Depressed *n* (%)	No depression *n* (%)	COR (95%CI)	*P*-value
Age	15–19	3 (23.1)	10 (76.9)	0.343 (0.083, 1.414)	0.139^∗∗^
	20–24	29 (20.4)	113 (79.6)	0.293 (0.144, 0.599)	0.001^∗^
	25–29	49 (21.2)	182 (78.8)	0.308 (0.158, 0.598)	0.001^∗^
	30–34	24 (21.8)	86 (78.2)	0.319 (0.152, 0.669)	0.002^∗^
	>35	21 (46.7)	24 (53.3)	1	

Residence	Rural	116 (24.7)	354 (75.3)	1.999 (0.992, 4.028)	0.053^∗∗^
	Urban	10 (14.1)	61 (85.9)	1	

Work status of women	Unemployed	120 (24.7)	366 (75.3)	2.678 (1.119, 6.407)	0.027^∗^
	Employed	6 (10.9)	49 (89.1)	1	

Educational status of women	Cannot read and write	28 (23.5)	91 (76.5)	2.110 (0.859, 5.184)	0.104^∗∗^
	Read and write	28 (41.2)	40 (58.8)	4.800 (1.897, 12.147)	0.001^∗^
	Primary	36 (27.5)	95 (72.5)	2.598 (1.077, 6.270)	0.034^∗^
	Secondary	27 (16.1)	141 (83.9)	1.313 (0.537, 3.209)	0.550
	Higher or tertiary	7 (12.7)	48 (87.3)	1	

Marital status of women	Others	54 (71.1)	22 (28.9)	13.398 (7.688, 23.356)	0.000^∗^
	Married	72 (15.5)	393 (84.5)	1	

Average family monthly income (Eth. birr)	<1000	107 (27.1)	288 (72.9)	3.344 (1.622, 6.894)	0.001^∗^
	1001–2000	10 (17.9)	46 (82.1)	1.957 (0.741, 5.164)	0.175^∗∗^
	>2001	9 (10)	81 (90)	1	

Parity	Primipara	40 (29.2)	97 (70.8)	1.663 (1.044, 2.650)	.032^∗^
	Multipara	59 (19.9)	238 (80.9)	1	

History of abortion	Yes	20 (45.5)	24 (54.5)	3.281 (1.725, 6.239)	0.000^∗^
	No	79 (20.3)	311 (79.7)	1	

History of still birth	Yes	20 (62.5)	12 (37.5)	6.814 (3.197, 14.524)	0.000^∗^
	No	79 (19.7)	323 (80.3)	1	

History of complication during last pregnancy, labor, and delivery	Yes	27 (45.8)	32 (54.2)	3.551 (2.002, 6.298)	0.000^∗^
	No	72 (19.2)	303 (80.8)	1	

Current pregnancy status planned	No	73 (57)	55 (43)	9.015 (5.729, 14.188)	0.000^∗^
	Yes	53 (12.8)	360 (87.2)	1	

History of previous depression	Yes	49 (46.2)	57 (53.8)	3.997 (2.537, 6.295)	0.000^∗^
	No	77 (17.7)	358 (82.3)	1	

Family history of mental illness	Yes	20 (66.7)	10 (33.3)	7.642 (3.473, 16.814)	0.000^∗^
	No	106 (20.7)	405 (79.3)	1	

Recent violence from intimate partner	Yes	37 (59.7)	25 (40.3)	6.485 (3.715, 11.322)	0.000^∗^
	No	89 (18.6)	390 (81.4)	1	

Marital satisfaction	Not satisfied	102 (67.1)	50 (32.9)	31.025 (18.190, 52.917)	0.000^∗^
	Satisfied	24 (6.2)	365 (93.8)	1	

Social support level	Poor	55 (50)	55 (50)	25.300 (12.141, 52.719)	0.000^∗^
	Moderate	61 (36.3)	107 (63.7)	14.423 (7.121, 29.215)	0.000^∗^
	Strong (good)	10 (3.8)	253 (96.2)	1	

Substance use by mother	Yes	12 (57.1)	9 (42.9)	4.749 (1.952, 11.550)	.001^∗^
	No	114 (21.9)	406 (78.1)	1	

Substance use by husband	Yes	27 (28.4)	68 (71.6)	1.392 (0.845, 2.291)	0.194^∗∗^
	No	99 (22.2)	347 (77.8)	1	

Note: ^∗^*p* value of less than 0.05, ^∗∗^*p* value of less than 0.25, and greater than 0.05 in bivariate analysis, “1” reference group.

**Table 4:** Factors associated with antenatal depression among antenatal mothers in West Badewacho Woreda, Hadiyya zone, South Ethiopia, 2018.

Variables	Category	Depressed N (%)	Not depressed N (%)	COR (95%CI)	AOR (95%CI)	*p*-value
Marital status	Others	54 (71.1%)	22 (28.9%)	13.398 (7.688, 23.356)	2.807 (1.268, 6.227)^∗^	0.011
	Married	72 (15.5%)	393 (84.5%)	1	1	

Current pregnancy status planned	No	73 (57%)	55 (43%)	9.015 (5.729, 14.188)	2.013 (1.025, 3.953)^∗^	0.042
	Yes	53 (12.8%)	360 (87.2%)	1	1	

History of previous depression	Yes	49 (46.2%)	57 (53.8%)	3.997 (2.537, 6.295)	3.414 (1.653, 7.052)^∗^	0.001
	No	77 (17.7%)	358 (82.3%)	1	1	

Family history of mental illness	Yes	20 (66.7%)	10 (33.3%)	7.642 (3.473, 16.814)	3.874 (1.154, 12.999)^∗^	0.028
	No	106 (20.7%)	405 (79.3%)	1	1	

Recent violence from intimate partner	Yes	37 (59.7%)	25 (40.3%)	6.485 (3.715, 11.322)	3.223 (1.359, 7.643)^∗^	0.008
	No	89 (18.6%)	390 (81.4%)	1	1	

Marital satisfaction level	Not satisfied	102 (67.1%)	50 (32.9%)	31.025 (18.190, 52.917)	7.568 (3.943, 14.523)^∗^	<0.001
	Satisfied	24 (6.2%)	365 (93.8%)	1	1	

Social support level	Poor	55 (50%)	55 (50%)	25.300 (12.141, 52.719)	5.491 (2.086, 14.451)^∗^	0.001
	Moderate	61 (36.3%)	107 (63.7%)	14.423 (7.121, 29.215)	5.788 (2.361, 14.138)^∗^	<0.001
	Strong (good)	10 (3.8%)	253 (96.2%)	1	1	

Note: ^∗^ Statistically significant association in multivariate analysis, “1” reference group.

## Data Availability

The datasets during the current study are available from the corresponding author on reasonable request.

## Abbreviations

CI: Confidence interval
EPDS: Edinburgh postnatal depression scale
SPSS: Statistical package for social sciences
